# Supplementary material for: Synergy of cations in high entropy oxide lithium ion battery anode
Source: Nat Commun. 2023 Mar 17;14:1487. doi: 10.1038/s41467-023-37034-6 (PMC10023782; doi:10.1038/s41467-023-37034-6)
Supplement: Supplementary file 1 — Supplementary Information [file 41467_2023_37034_MOESM1_ESM.pdf]

Supplementary Information File for

Synergy of cations in high entropy oxide lithium ion battery anode

Kai Wang[1,2], Weibo Hua[3], Xiaohui Huang[1,2], David Stenzel[1,2], Junbo Wang[1,2], Ziming Ding[1,2], Yanyan Cui[1,2], Qingsong Wang[1], Helmut Ehrenberg[3], Ben Breitung[1,2], Christian Kübel[1,2,4,5]*, Xiaoke Mu[1]*

1 Institute of Nanotechnology (INT), Karlsruhe Institute of Technology, Hermann-von-Helmholtz-Platz 1, 76344 Eggenstein-Leopoldshafen, Germany;

2 Department of Materials and Earth Sciences, Technical University Darmstadt, 64287 Darmstadt, Germany;

3 Institute for Applied Materials (IAM), Karlsruhe Institute of Technology, Hermann-von-Helmholtz-Platz 1, 76344 Eggenstein-Leopoldshafen, Germany;

4 Helmholtz-Institute Ulm for Electrochemical Energy Storage (HIU), Karlsruhe Institute of Technology (KIT), Helmholtzstraße 11, 89081 Ulm, Germany;

5 Karlsruhe Nano Micro Facility (KNMF), Karlsruhe Institute of Technology (KIT), Hermann-von-Helmholtz-Platz 1, 76344 Eggenstein-Leopoldshafen, Germany.

Corresponding: xiaoke.mu@kit.edu and christian.kuebel@kit.edu

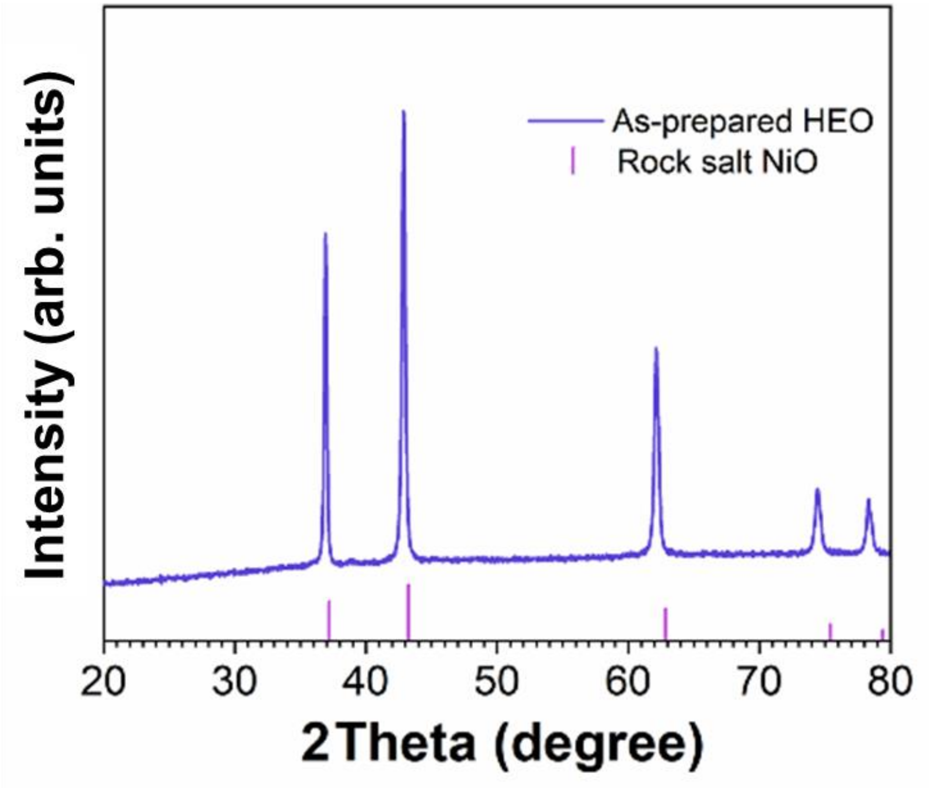

Supplementary Figure 1. The XRD pattern of as-prepared $Mg_{0.2}Co_{0.2}Ni_{0.2}Cu_{0.2}Zn_{0.2}O$ HEO.

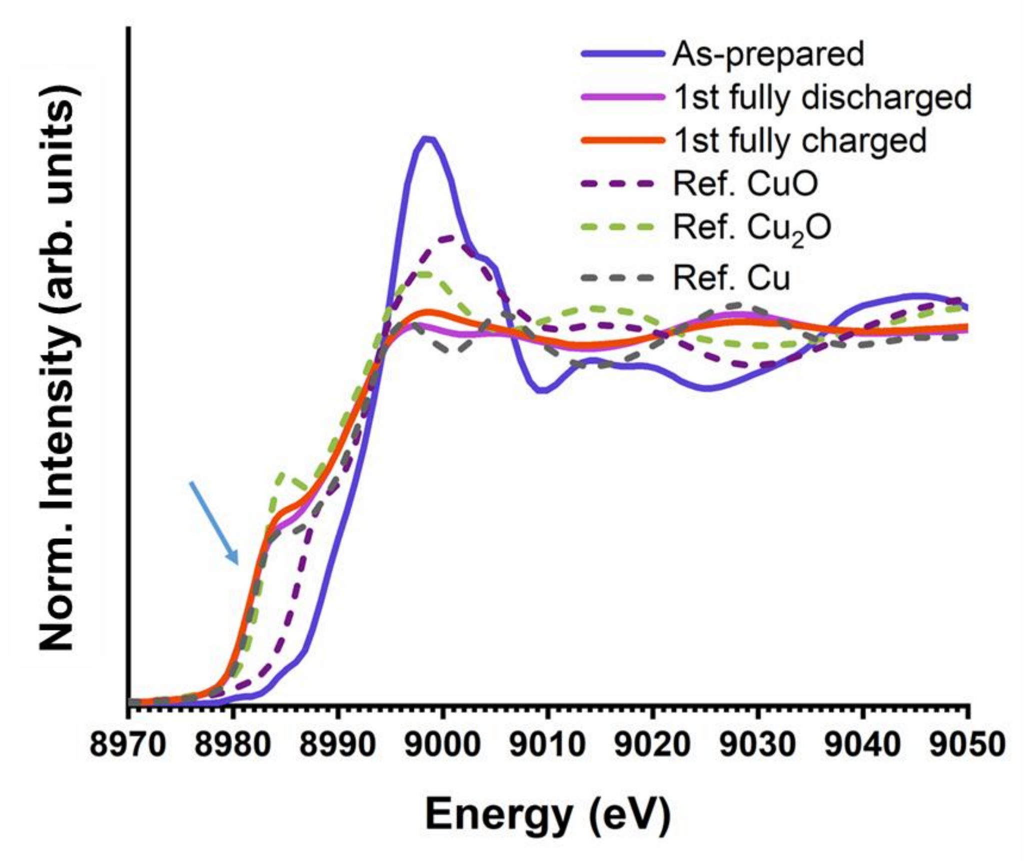

Supplementary Figure 2. The Cu K-edge of the XANES spectrums of as-prepared and cycled HEO samples and of CuO, Cu_2O and metallic Cu as references. Looking at the near edges of CuO, Cu_2O and Cu references, the clear pre-edges in the spectra of metallic and Cu^{1+} can be observed. (marked by arrow) Although the onset positions in as-prepared sample and CuO reference are imperfect overlapped, the worse consistence could be found among the experiment data (as-prepared HEO) and the references of Cu_2O and metallic Cu. The shape of XANES could be affected by structure/element environment[1], the difference between the spectrums of experiment and reference should be caused by the divergent crystal structures between the rock-salt of HEO and tenorite structure for CuO reference.

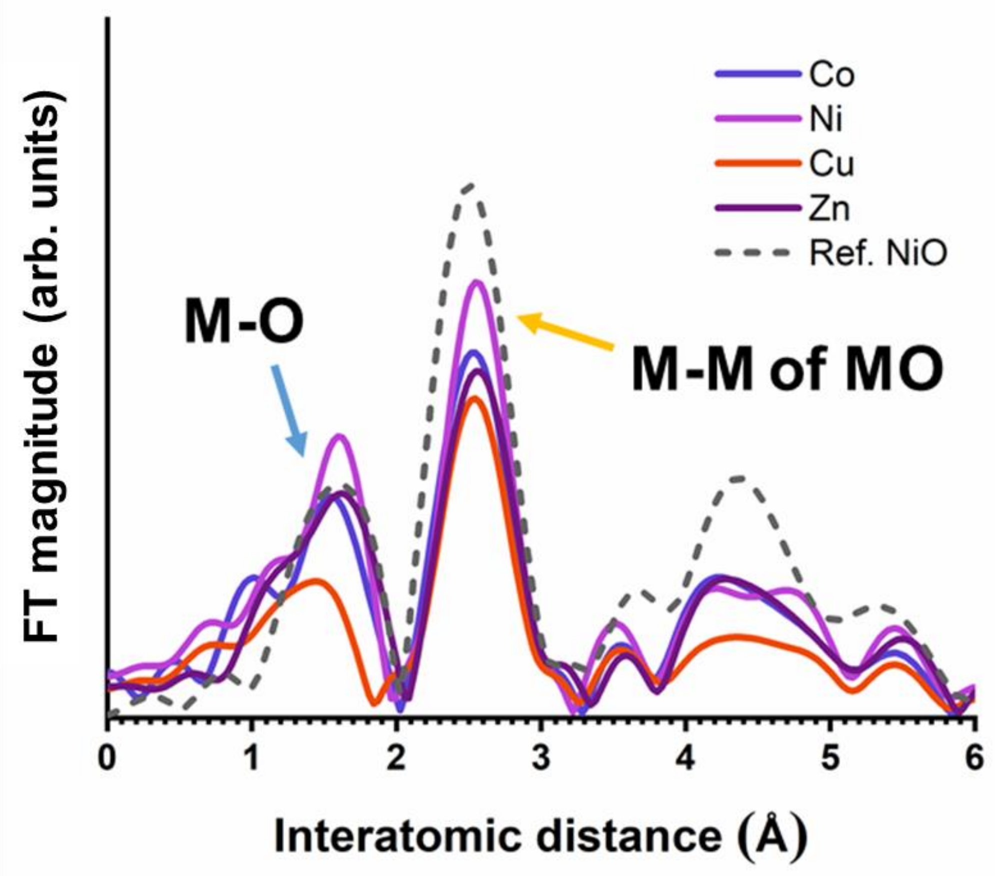

Supplementary Figure 3. The superposition of EXAFS-FT spectra of Co, Ni, Cu and Zn in the as-prepared sample. The EXAFS-FT spectra show the well-presented metal-oxygen (M-O) shells and metal-oxygen-metal (M-O-M) shells (i.e., interatomic distance) locating at the same position of the shells of the NiO reference. This indicates that all kind metal elements have the identical M-O bonding length in the as-prepared sample (the spectra are superpositioned for eye guidance), and confirms the rock salt type face-centered cubic (fcc) structure of the HEO, in agreement with the previous XRD result.[2]

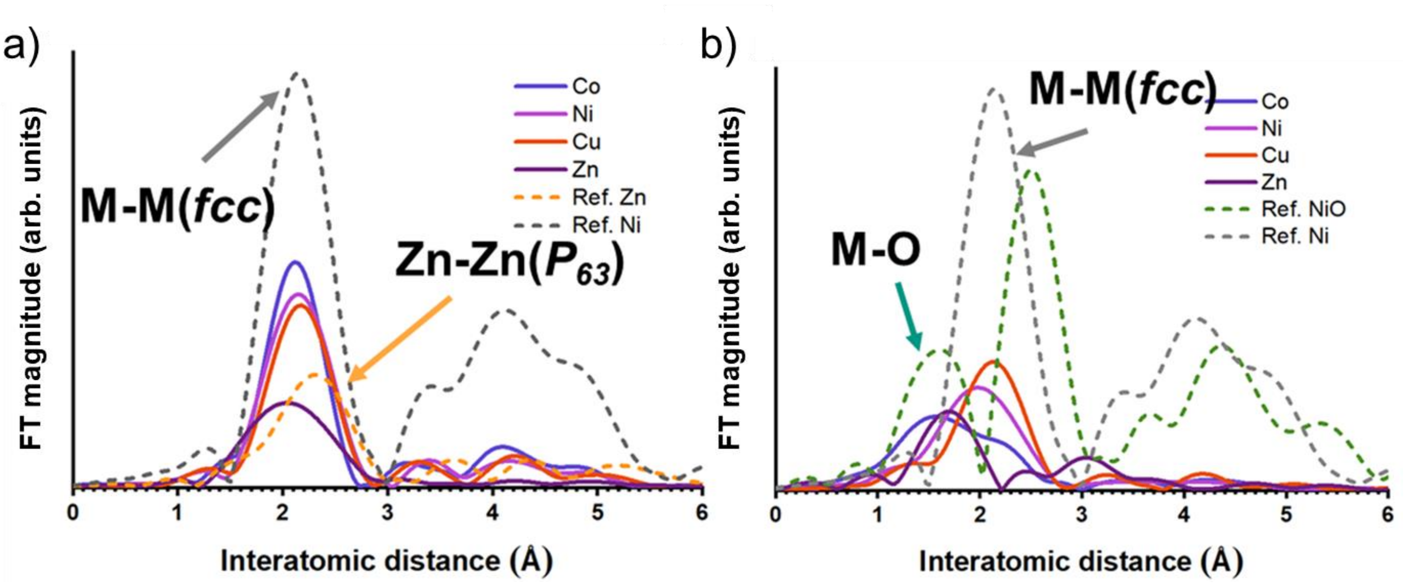

Supplementary Figure 4. The superposition of EXAFS-FT spectra of Co, Ni, Cu and Zn in the discharged sample (a)) and charged sample (b)). As described in the manuscript, the difference between the experimental data and the hexagonal (P63/ mmc) Zn metal structure could be explained by Zn alloying with Li generating an fcc ZnLi structure. Another possibility is that Zn forms an fcc alloy with some reduced transition metal elements, Co, Ni and Cu, at the discharged state. However, as shown in Supplementary Figure 4a, Zn shows a weaker and broader shell shape compared with the other three elements. It implies the Zn could be in a different elemental environment with the others, which does not fit to Zn forming an alloy with the other transition metal elements.

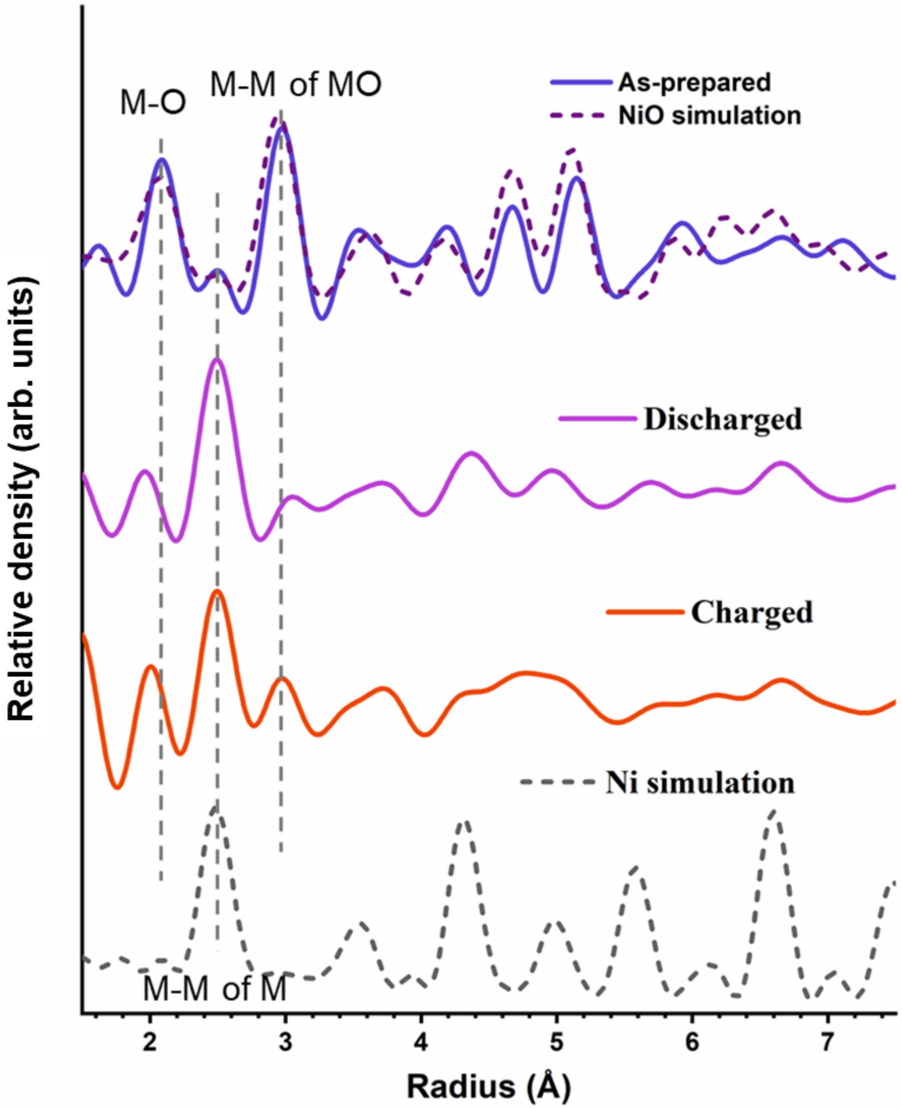

Supplementary Figure 5. ePDFs for the as-prepared (blue solid line), discharged (pink solid line) and charged sample (red solid line), compared to simulated ePDFs (dashed lines) based on the crystal structure of NiO (rock-salt)[3] and Ni (fcc)[4]. The atomic structure of the dis/charged samples was investigated using electron pair distribution function (ePDF) analysis derived from select area electron diffraction (SAED) patterns. The ePDF of the as-prepared HEO confirms the rock-salt structure. The M-O-M peak at 3.0 Å and the M-O peak at 2.0 Å, which were highly pronounced in the as-prepared sample, disappeared or are significantly reduced in the ePDFs of the discharged and recharged samples, whereas a new peak at 2.5 Å corresponding to M-M bonding in fcc metals appeared. This provides additional confirmation for the formation of the fcc metallic phase/phases in the dis/recharged samples deduced from the XAS analysis.

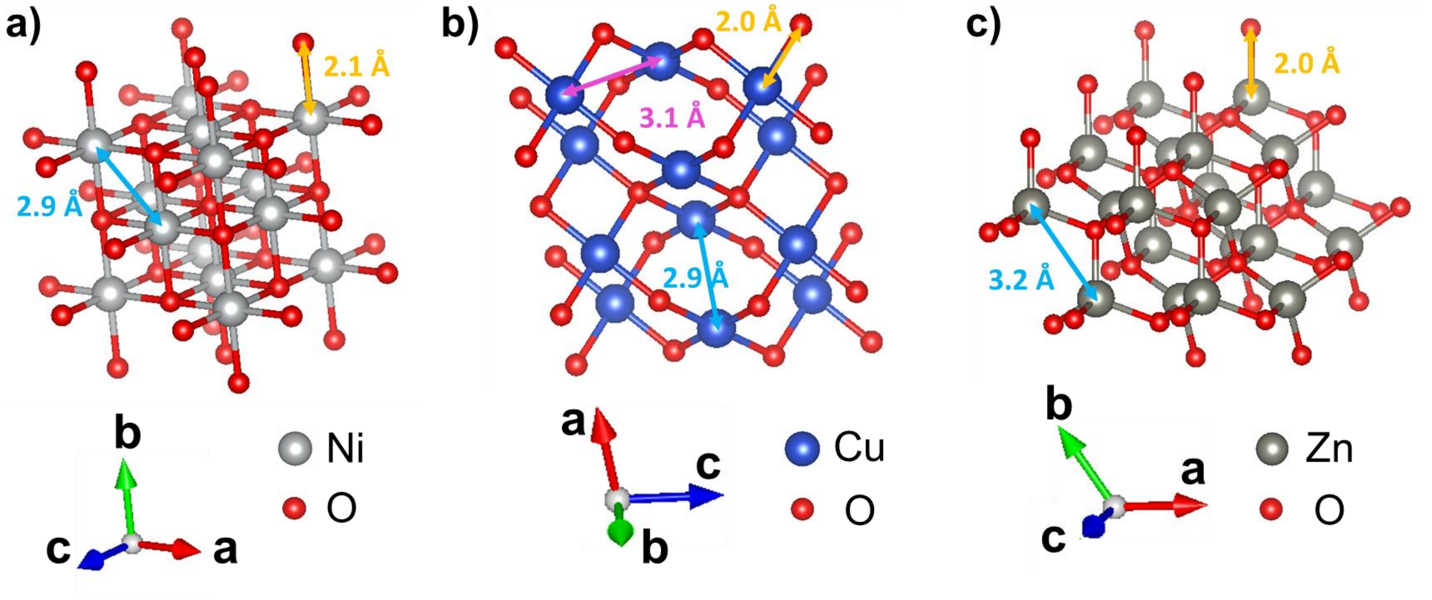

Supplementary Figure 6. The sketch of the atomic structures of rock-salt fcc NiO (a)), tenorite monoclinic crystal CuO (b)) and wurtzite hexagonal crystal ZnO (c)), the images were created from VESTA software. HEO has a rock-salt structure and its cell parameters are very close to NiO. The five metal elements (Mg, Co, Ni, Cu and Zn) take the equivalent position of the Ni atoms in the NiO structure. Therefore, we used NiO as the reference to compare to the experimental EXAFS-FT data of Co, Ni, Cu and Zn in Supplementary Figure 3. CuO and ZnO are tenorite monoclinic and wurtzite hexagonal structure as shown in Supplementary figure 6. The M-M and M-O distance in CuO and ZnO are different to them of NiO, CoO and MgO. However, the observation in the EXAFS-FT (Supplementary Figure 3) indicates that the M-O and M-O-M shells of the Cu and Zn atoms in the as-prepared sample match very well to the corresponding shells of the rock-salt structured NiO, CoO and MgO. Therefore, Cu and Zn atoms in the as-prepared sample join into the rock-salt structure forming the HEO configuration.

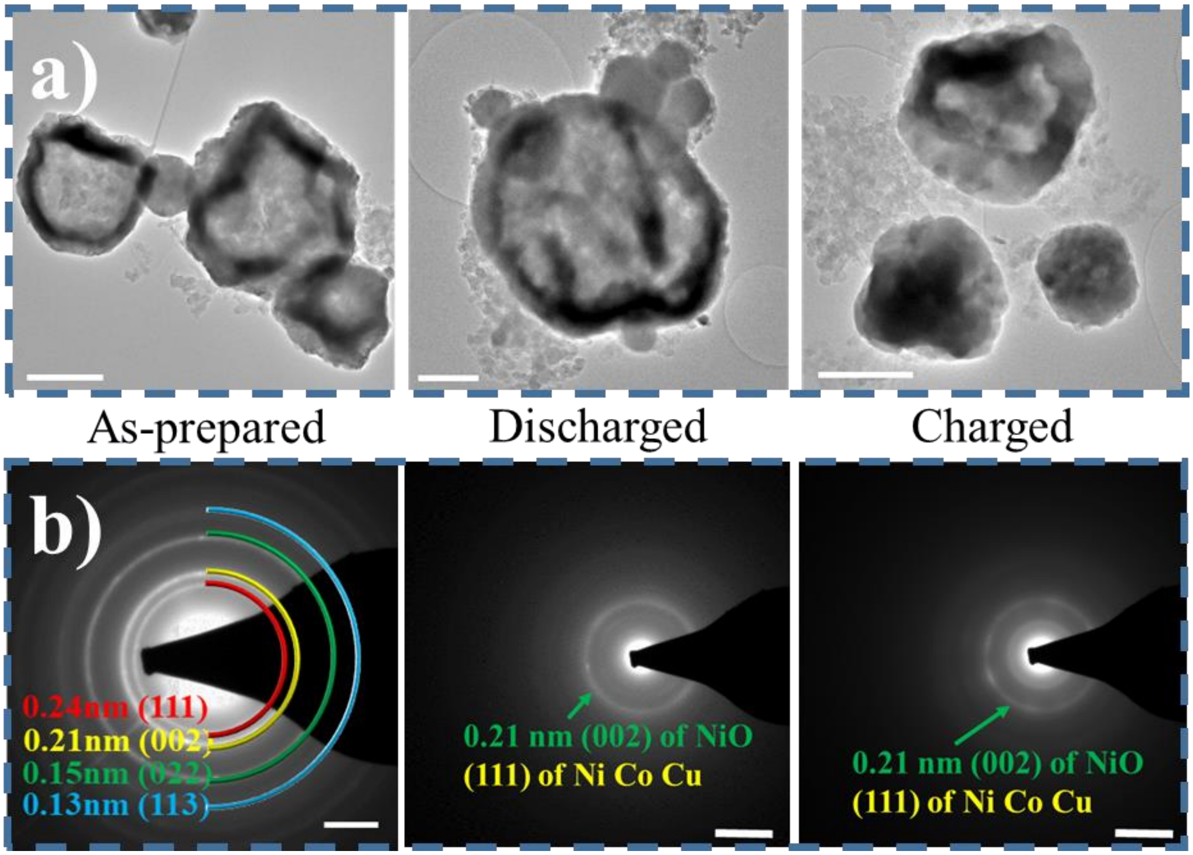

Supplementary Figure 7. a) and b) TEM images and SAED patterns of the as-prepared HEO, the discharged and charged samples, the scale bar in a) and b) are 0.5 μm and 5 1/nm, respectively. The discharged and charge sample show similar morphology with the as-prepared sample. The hollow spherical particles are not fragmented during cycling. The rings in the SAED pattern of the as-prepared sample (Supplementary Figure 5b) can be straightforwardly indexed to (111), (002), (022) and (113) lattices of the rock-salt structure. The structure disorders during cycling, in the discharged and charged samples only a few diffraction rings with significantly diffused intensity are found, rendering a fundamental difficulty to index the diffraction rings. For example, the major diffraction ring located at 0.21 nm can be indexed either to (002) lattices of the residual HEO structure or to metal Ni/Co/Cu (111) lattices.

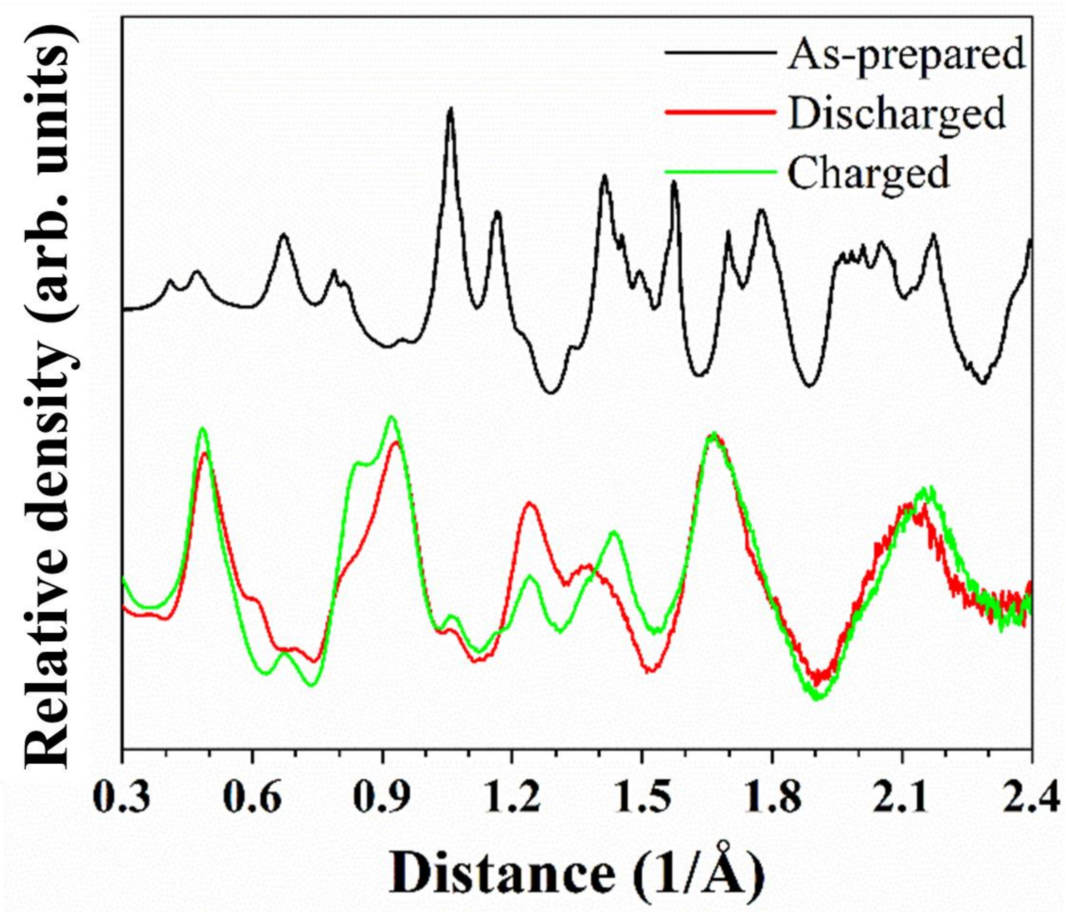

Supplementary Figure 8. Structure factor of the as-prepared, discharged and charged samples. The structure factor abstracted from the SAED pattern clearly reveals a big structure difference between the as-prepared and dis/charged samples, and the close similarity between the discharged and charged samples. This reveals that the re-charged sample does not convert back to HEO, but in a mixed state between metallic and oxide nanophases.

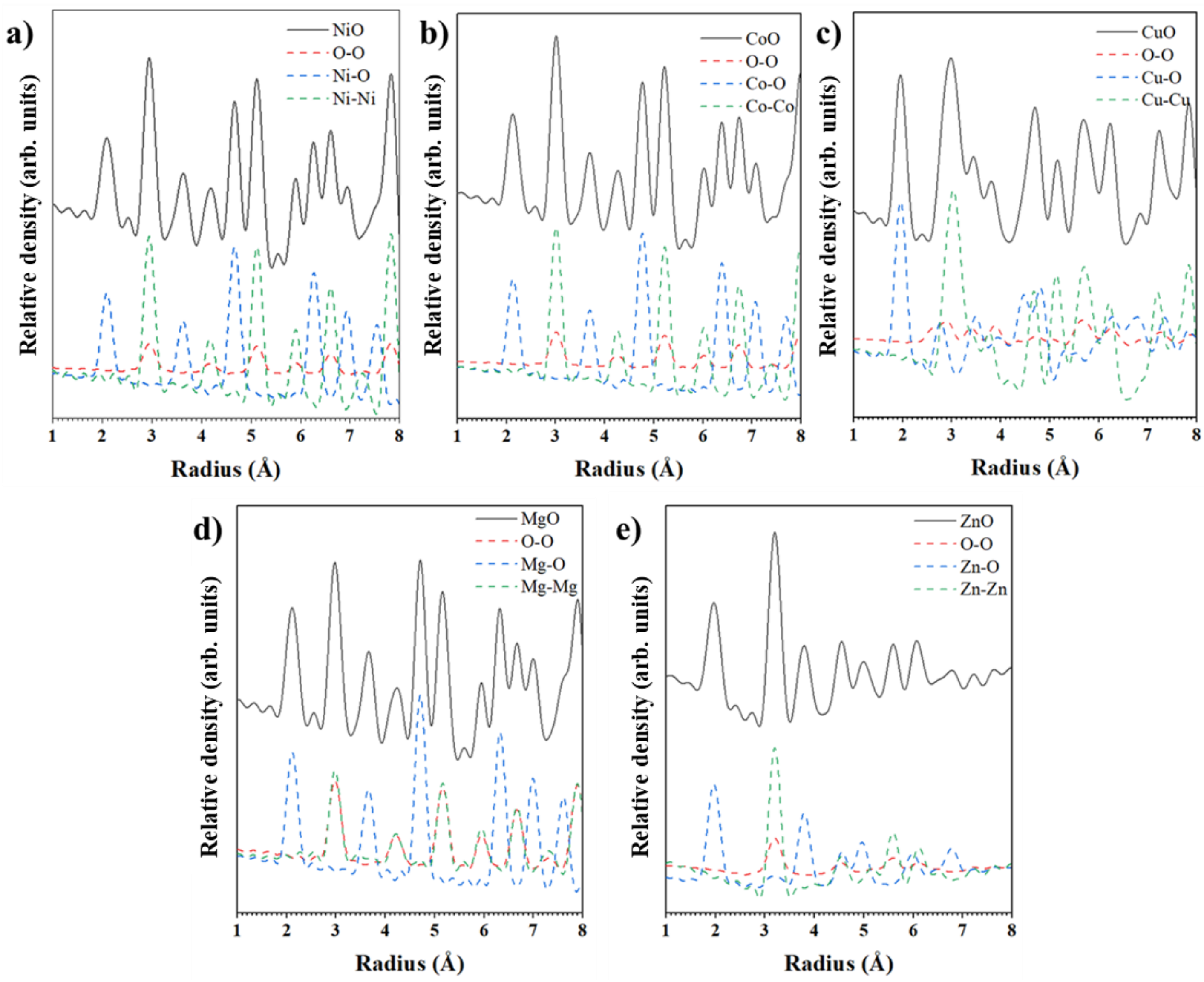

Supplementary Figure 9. Simulated RDF curves and corresponding partial RDF of NiO (a)), CoO (b)), CuO (c)), MgO (d)) and ZnO (e)).

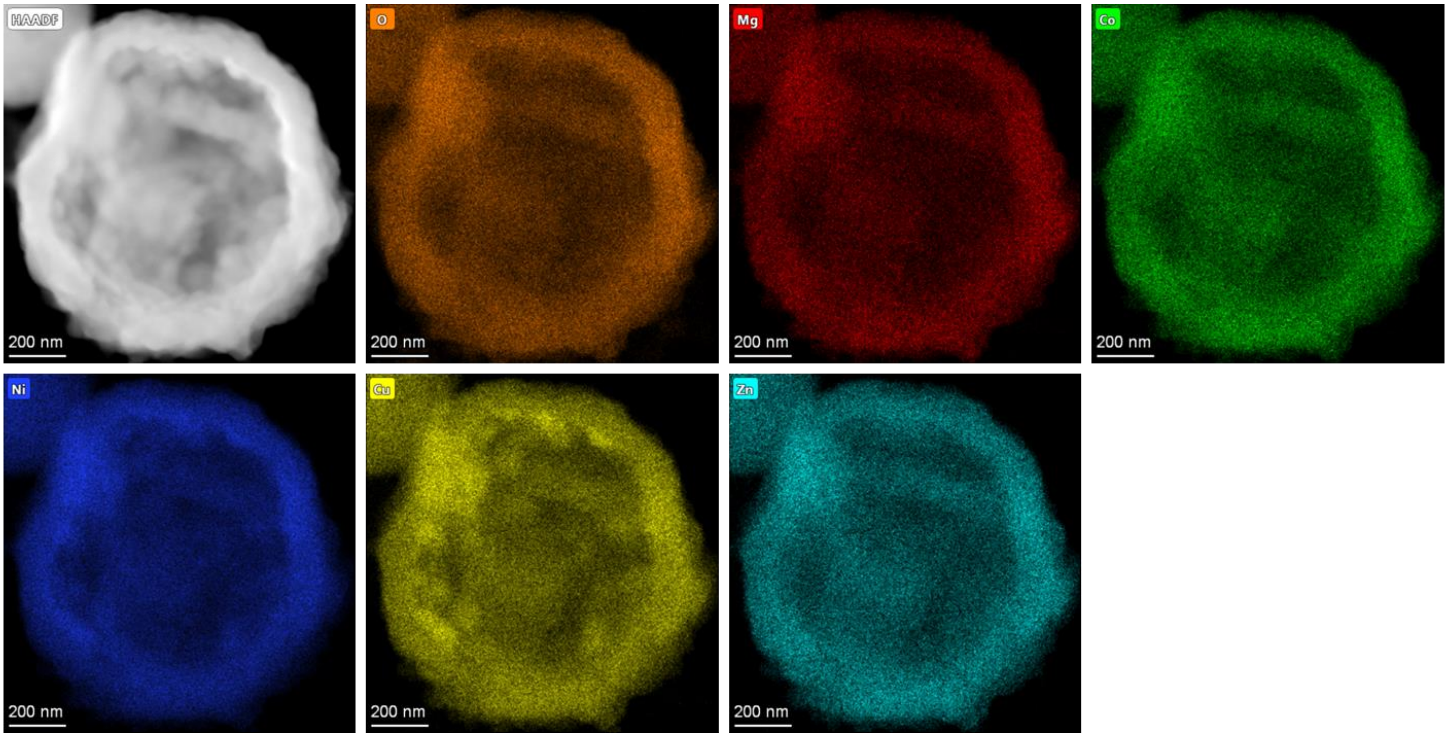

Supplementary Figure 10. STEM-EDX map of a complete particle in the charged state without FIB preparation. STEM-EDX was conducted to study the elements distribution in the bulky particles in the 1st charged sample. The pixel size is 3.3 nm. All the elements show homogeneous distributions. The result here is in consistent with our previous work reported in[2], however, it cannot deliver the accurate information of the elemental distribution because of the serious overlapping of the nanophases along the electron beam.

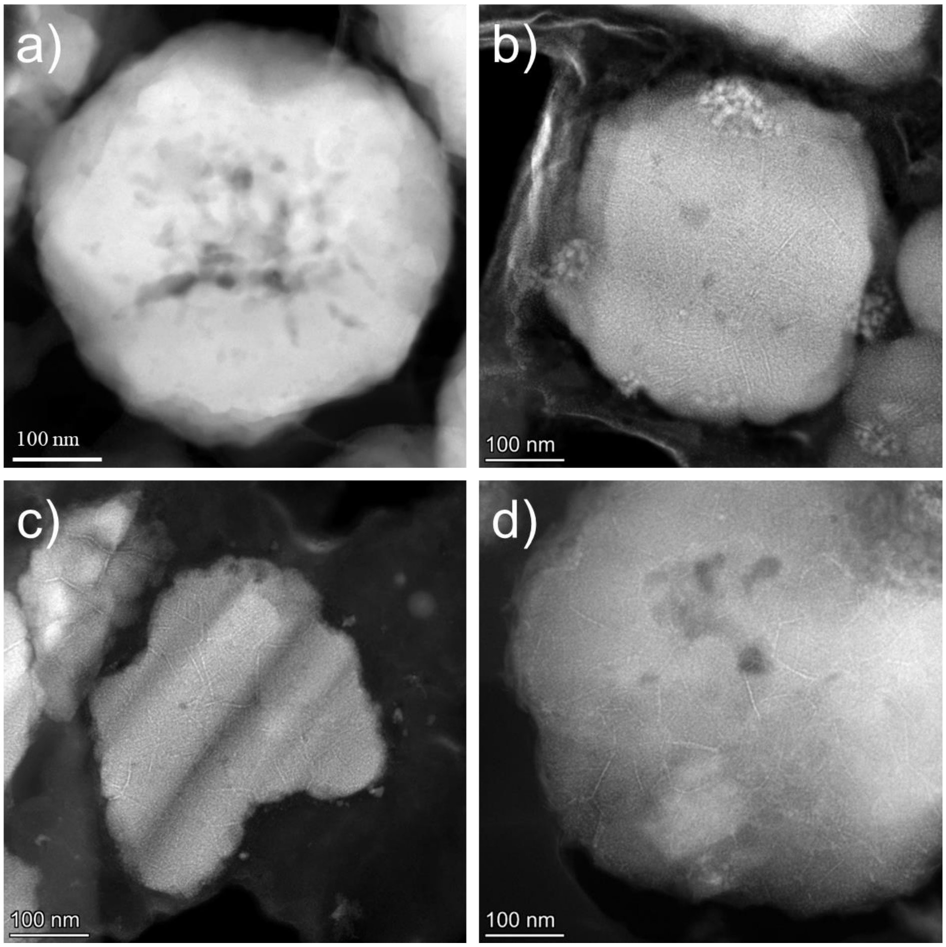

Supplementary Figure 11. STEM-HAADF images of the as-prepared FIB sample (a)), the 1st discharged FIB sample (b)), the 1st charged FIB sample (c)) and the 1st discharged ultramicrotomy sample (d)). Using FIB and ultramicrotome preparing thin samples significantly reduces he overlapping issue. Many details can be resolved. Supplementary Figure 11 shows the cycled samples contains the dendrites and inhomogeneous intensity significantly different from the as-prepared sample. Ultramicrotomy is a physical cutting method. The materials were embedded in resin followed by a diamond knife slicing using a step size of 70 nm. The identical behavior of the observed features between the FIB sample and ultramicrotome sample indicates that the dendrites at the grain boundary and the nanoscale spinodal fine structures inside the grains are not introduced by the ion beam during the FIB sample preparation.

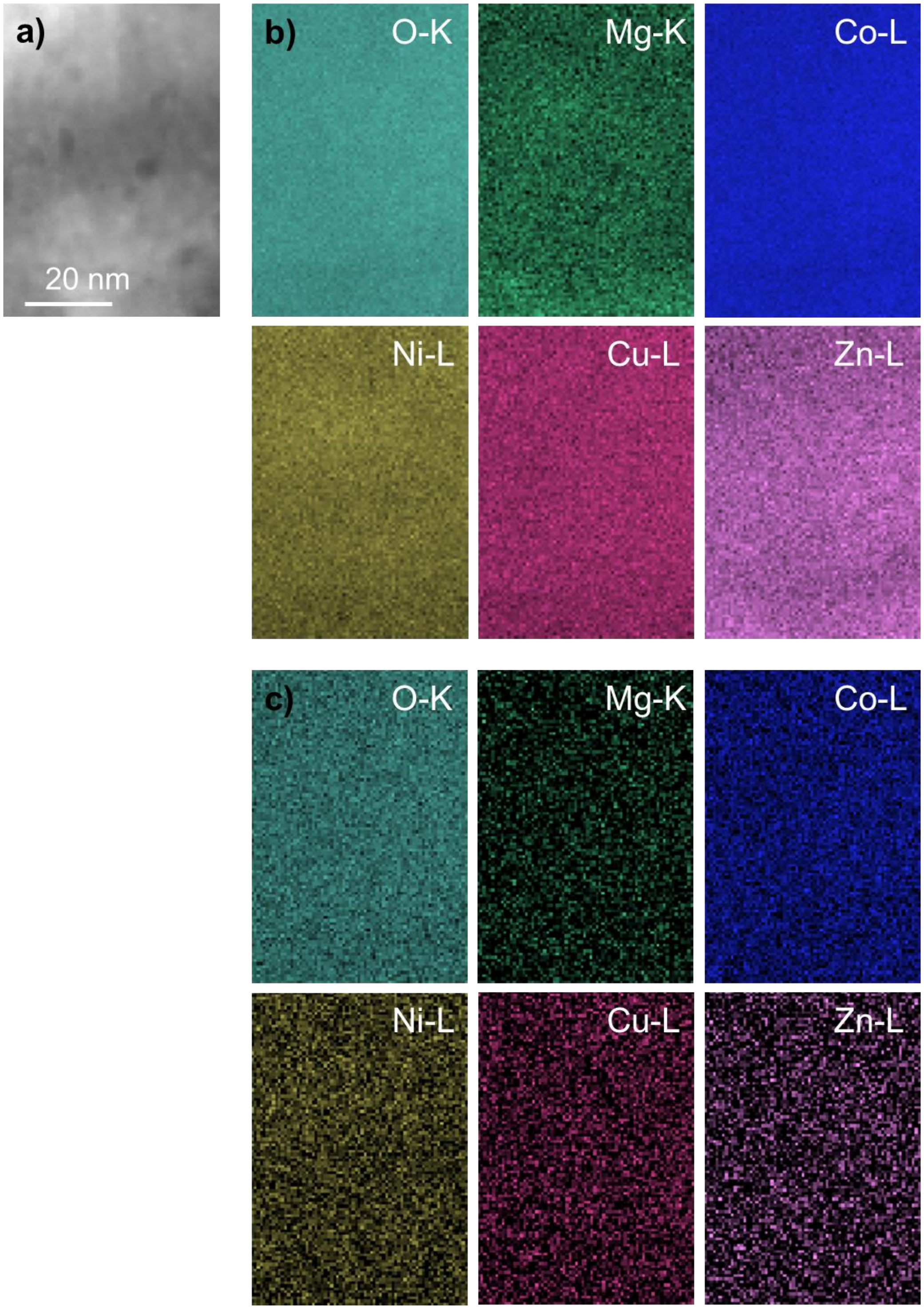

Supplementary Figure 12. The ADF image and EELS elemental maps of the as-prepared sample. a) ADF image; b) EELS elemental maps obtained after denoising process; c) elemental maps obtained from the non-denoised raw data.

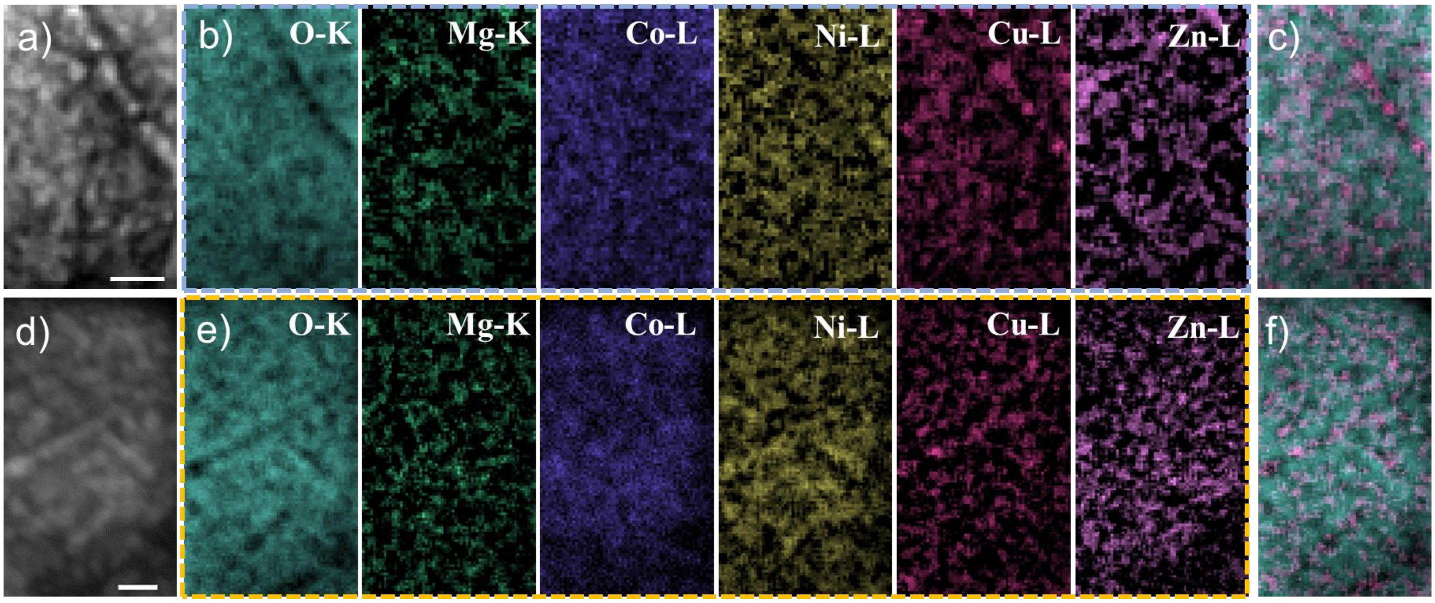

Supplementary Figure 13. The EELS map of the 1st discharged sample (a) to c)) and the 1st charged sample (d) to f)) obtained from the non-denoised raw data used for the Figure 2. a) and d) ADF images, the scale bars in a) and d) are 10 nm; b) and e) maps of each individual element. c) and f) the color mixed images of the O and Cu maps.

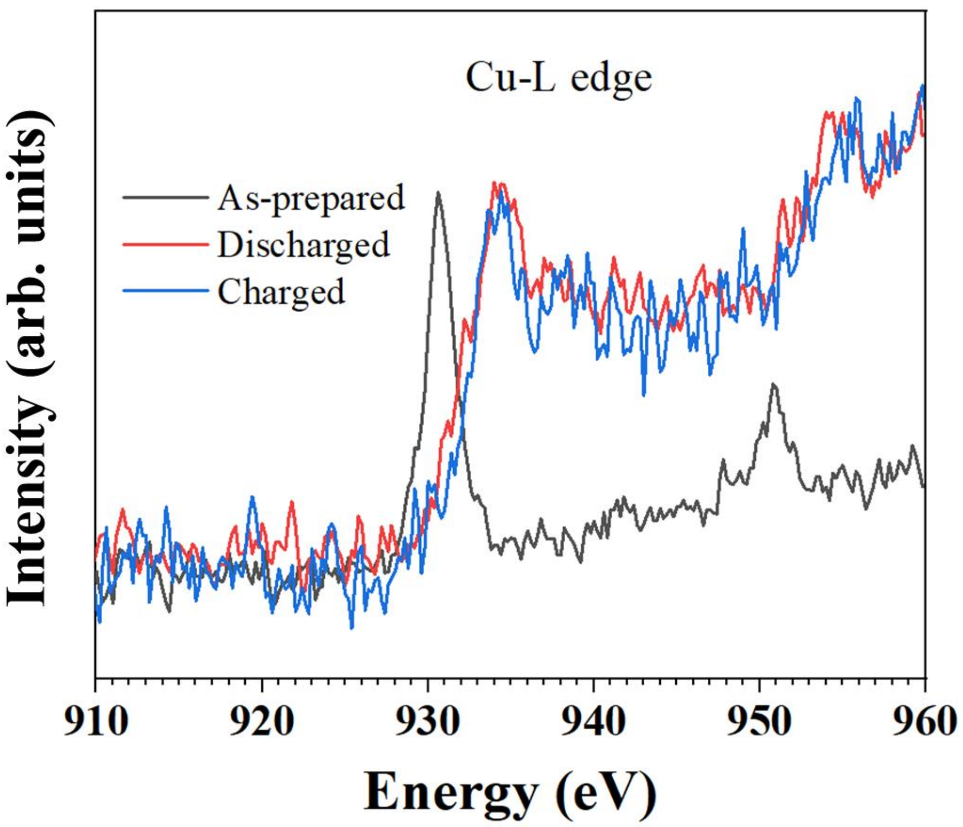

Supplementary Figure 14. Cu-L edge EELS spectra of the as-prepared and cycled samples. The Cu-L edges of the as-prepared and cycled samples show distinct difference that L3 and L2 edges were well presented in the as-prepared sample indicating an oxidized state, which cannot be found in the cycled samples. The white line (L3 and L2 edges) appeared in oxidized state is caused by the incompletely occupied 3d orbital. However, the 3d orbital is completely occupied in the metallic Cu leading to the missing of the white line.

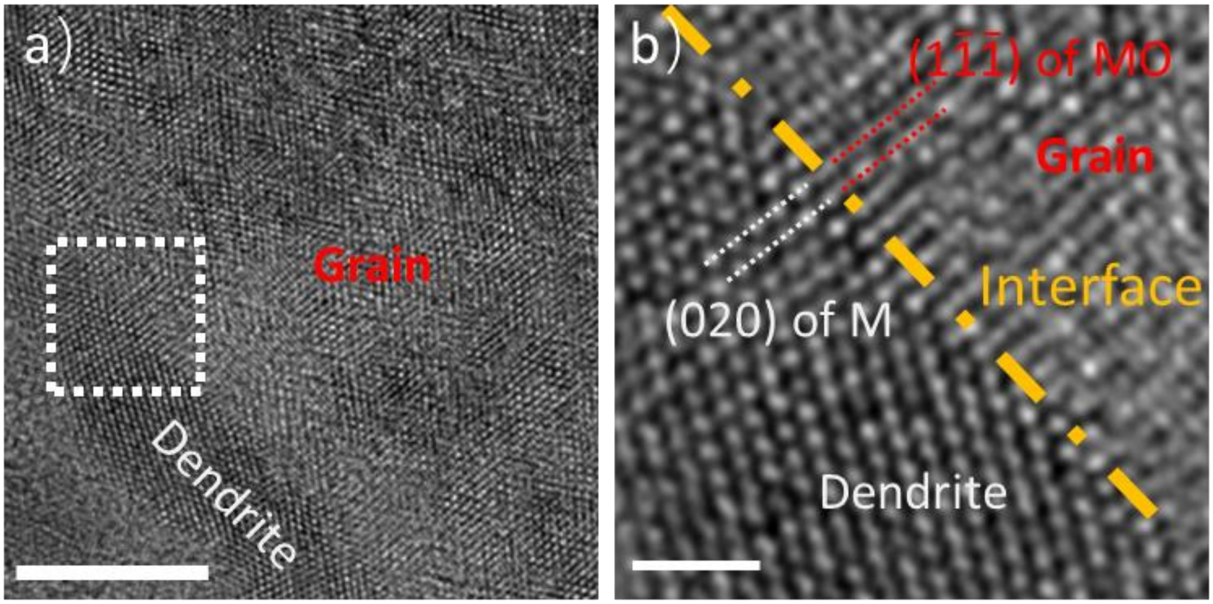

Supplementary Figure 15. a) HRTEM image of 1st charged sample, the scale bar is 5 nm; b) enlarged image of the marked area by white dash box in a), the scale bar is 1 nm.

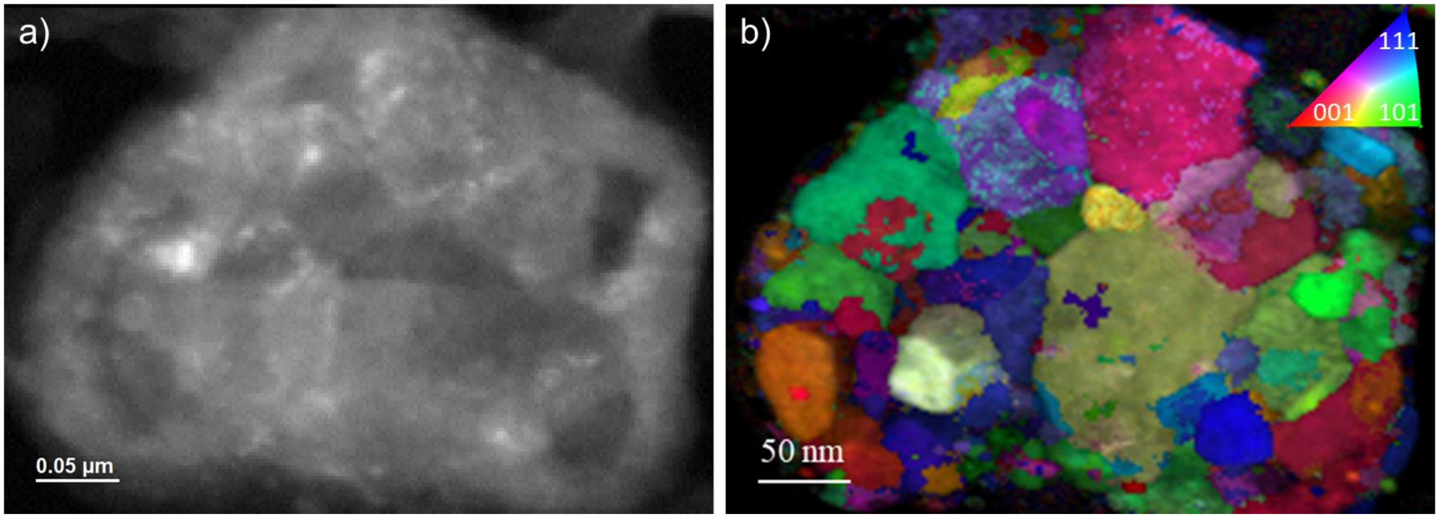

Supplementary Figure 16. 4D-STEM results of the as-prepared FIB sample. a) virtual HAADF image of the area of the 4D-STEM measurement of as-prepared sample, b) orientation map obtained from the 4D-STEM data. This figure shows the orientation map of the as-prepared sample. The polycrystalline structure of the sample with grain size spanning from tens to hundred nanometers is consistent with that in discharged sample (Figure 3). This reveals that the grains were preserved during the half-cell cycling.

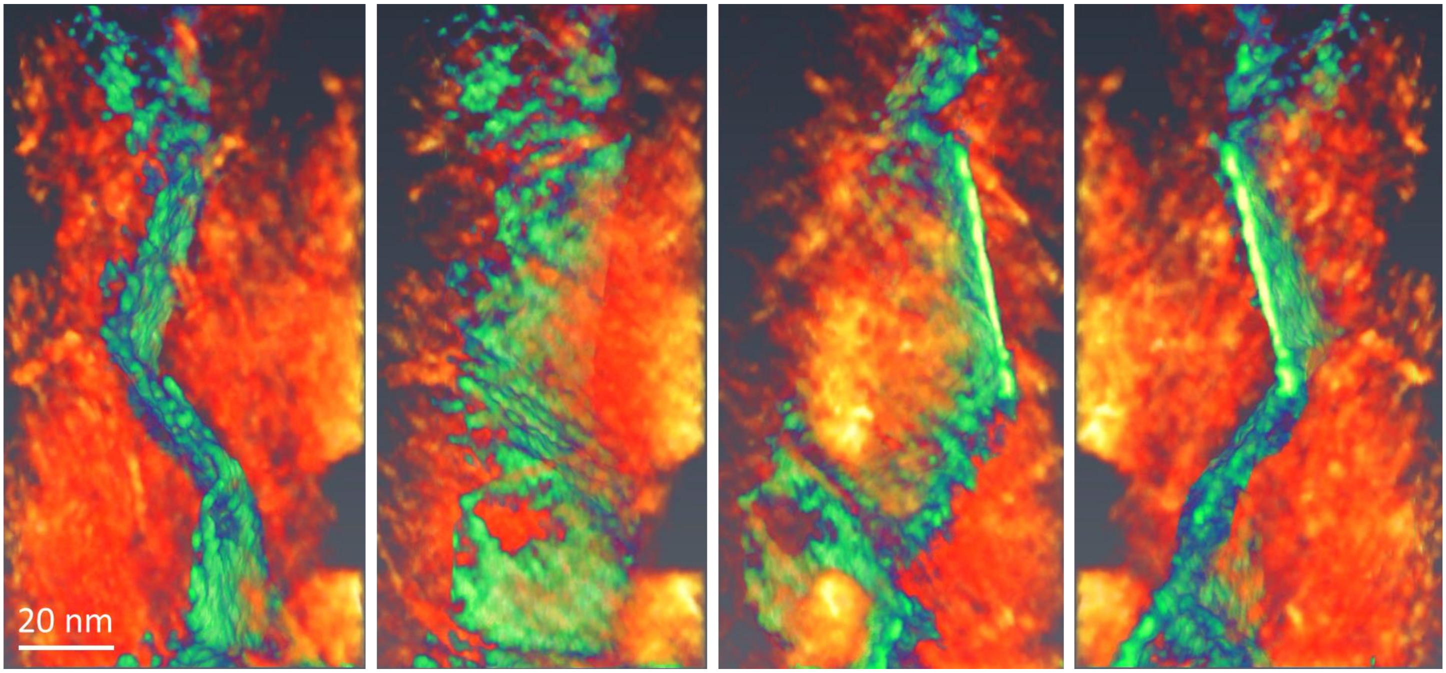

Supplementary Figure 17. A series of 3D tomography images from different viewing directions after combining the volume renderings of the grain boundary and surrounding region.

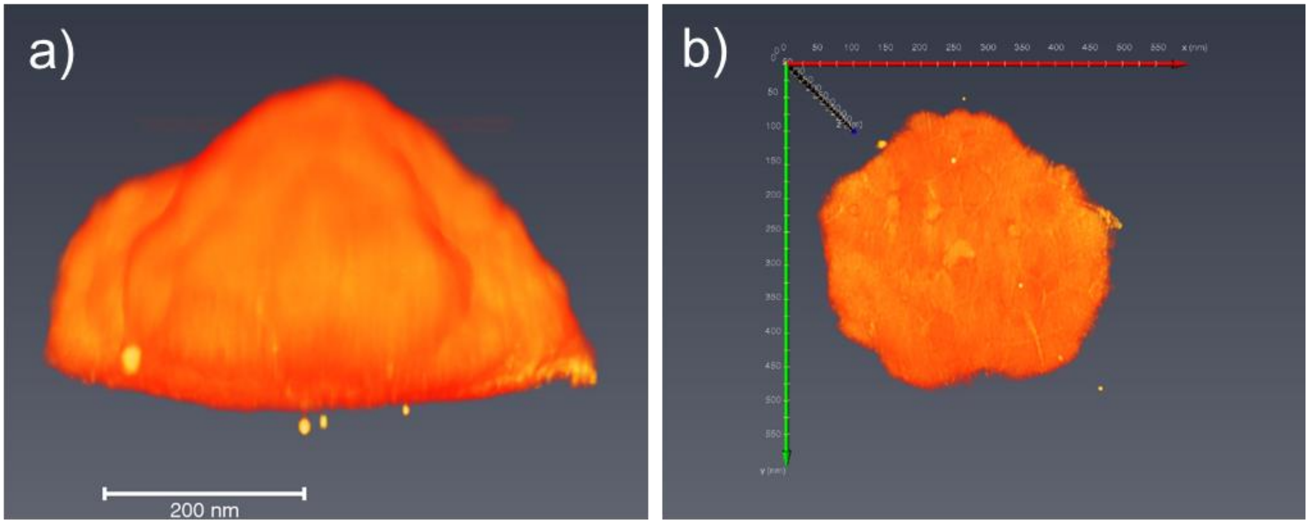

Supplementary Figure 18. Overview morphology of the particle used for the 3D morphology study. a) side view; b) front view of the reconstructed volume.

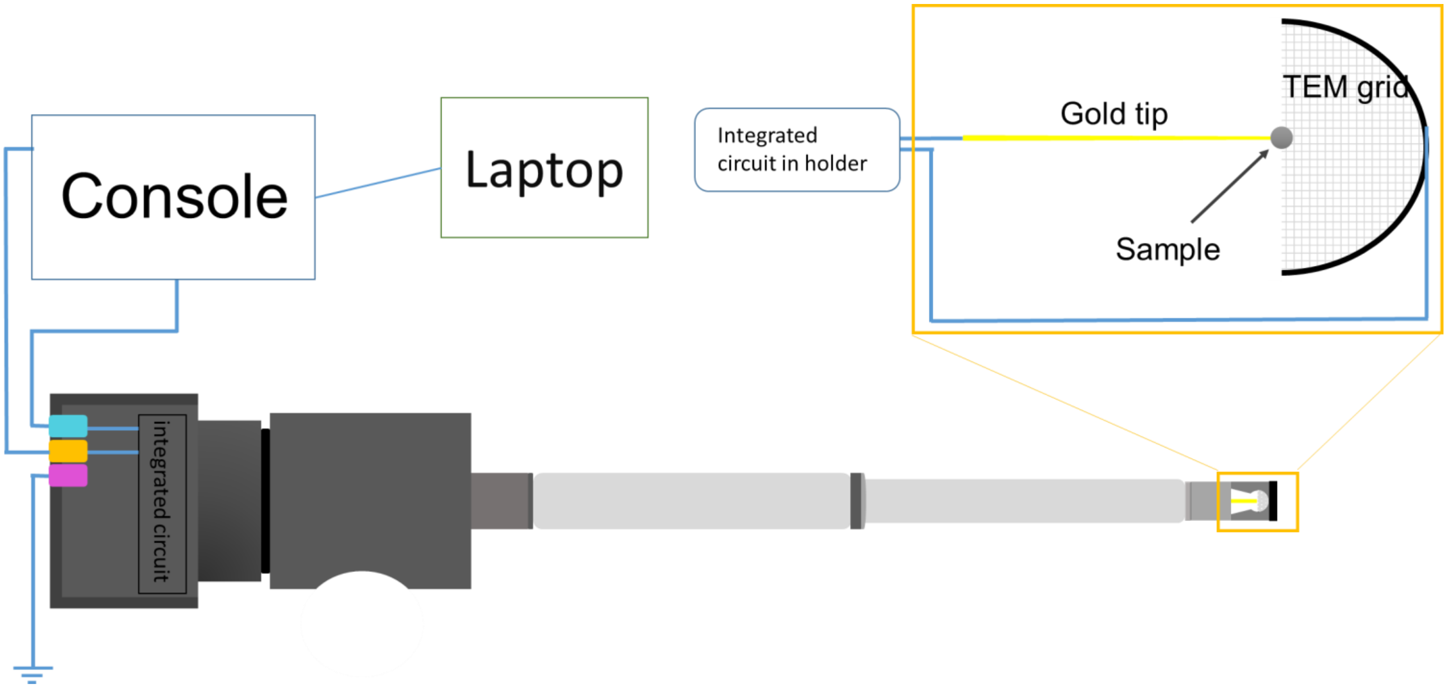

Supplementary Figure 19. The sketch of the STM holder and the working principle of the in-situ conductivity measurement. The STM tip made by gold wire with nanometer sharpness can move in x, y and z directions controlled by piezoelectric motor operated by the lab laptop. Sample particles are dispersed on a half TEM grid mounted on the other side of the holder slot. The STM tip and the sample grid are connected to an integrated circuit inside the holder, through which controlled voltage bias can be applied. Under STEM mode, the gold tip was controlled to touch the sample on the TEM grid with cautions to avoid any conductive substrates. After approached to the touch, an external voltage was applied in step of 0.01 V/s scanning from -15 V to 15 V.

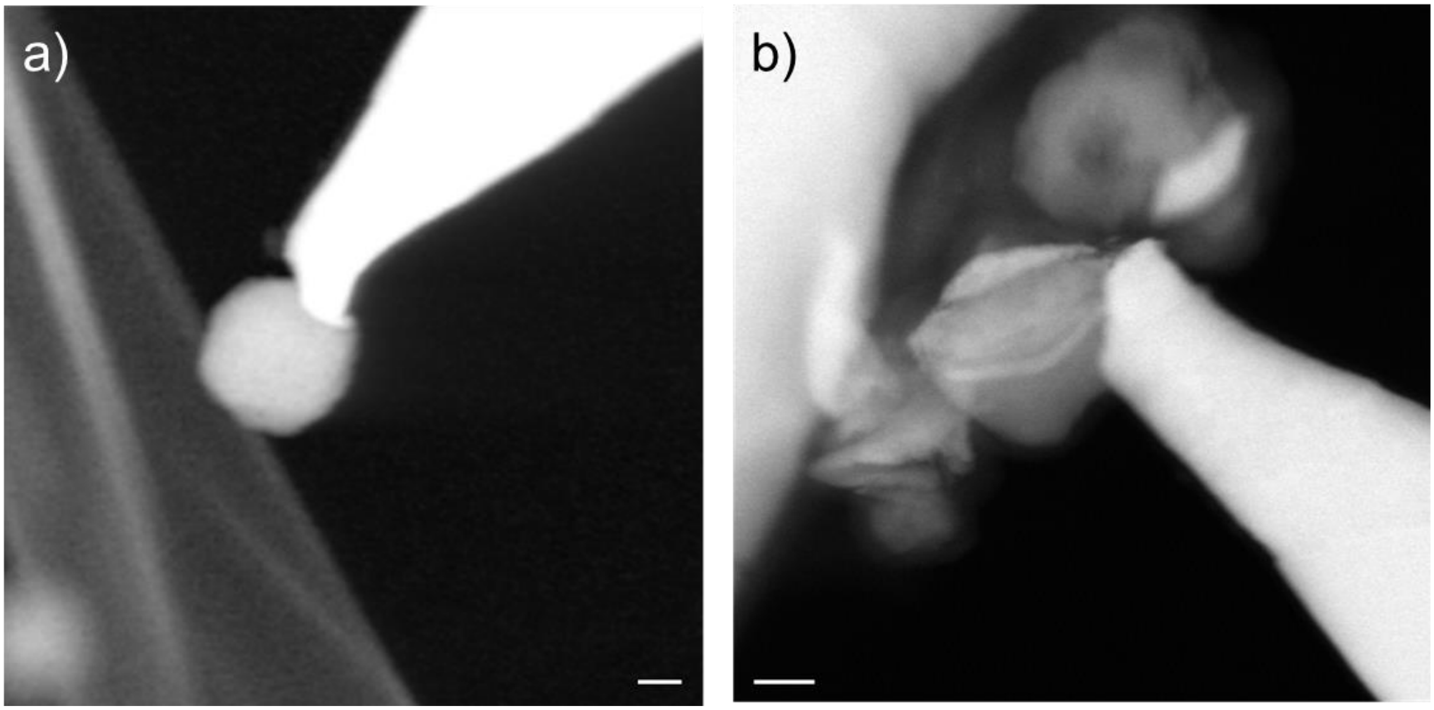

Supplementary Figure 20. The image of that the gold tip touches the samples. a) as-prepared sample, the scale bar is 100 nm; b) 1st charged sample, the scale bar is 100 nm.

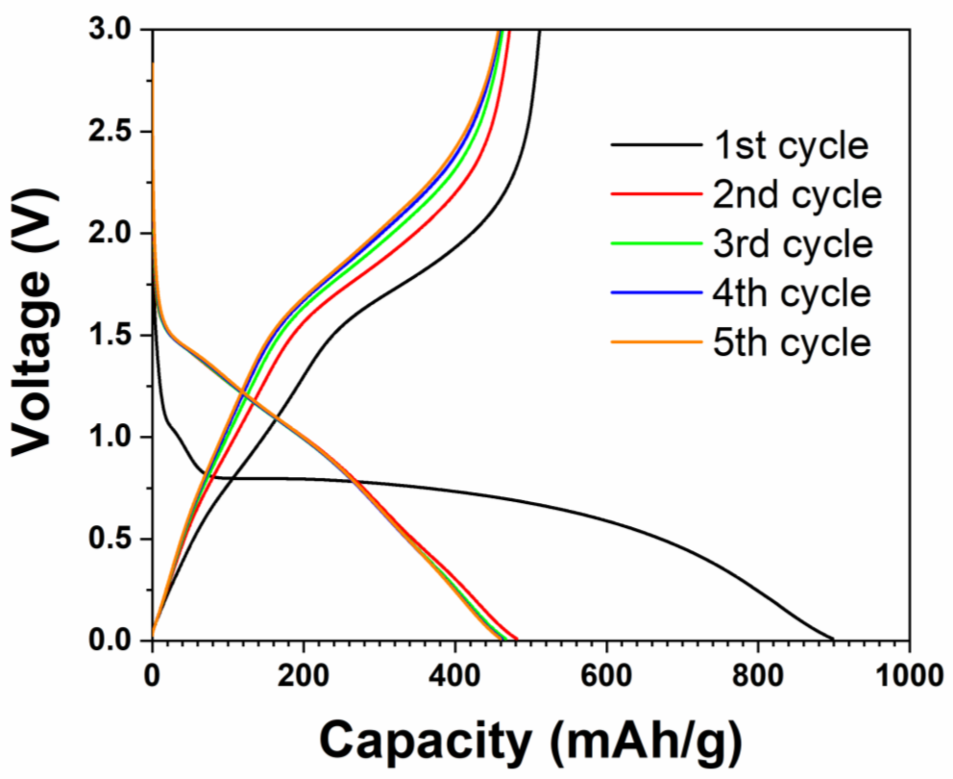

Supplementary Figure 21. The voltage-capacity profiles during the 1st to 5th electrochemical cycling reveal a good reversibility after the 1st cycle. The difference between the 1st discharge capacity and the discharge capacity in the following cycles can mainly be attributed to the irreversible oxide reduction and SEI formation.[5] Form the 1st recharging to 5th recharging, the capacity do not change significantly indicating reversible electrochemical processes in following cycles.

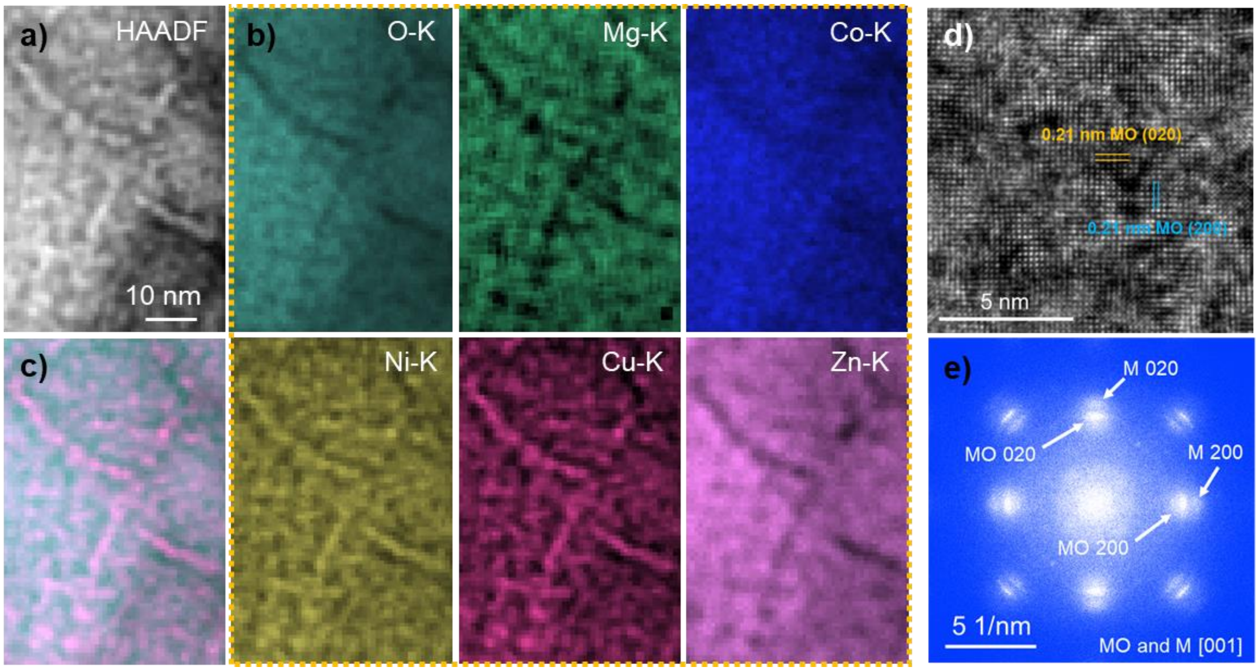

Supplementary Figure 22. (S)TEM, elemental distribution and crystal structure analysis of a sample after 5 cycles showing very similar features as seen previously after the first cycle. a) HAADF-STEM image of the sample after the 5th recharging; b) EELS elemental maps; c) combined O and Cu maps; d) HRTEM (enlarged section from Supplementary Figure 23); e) FFT of the image shown in Supplementary Figure 23.

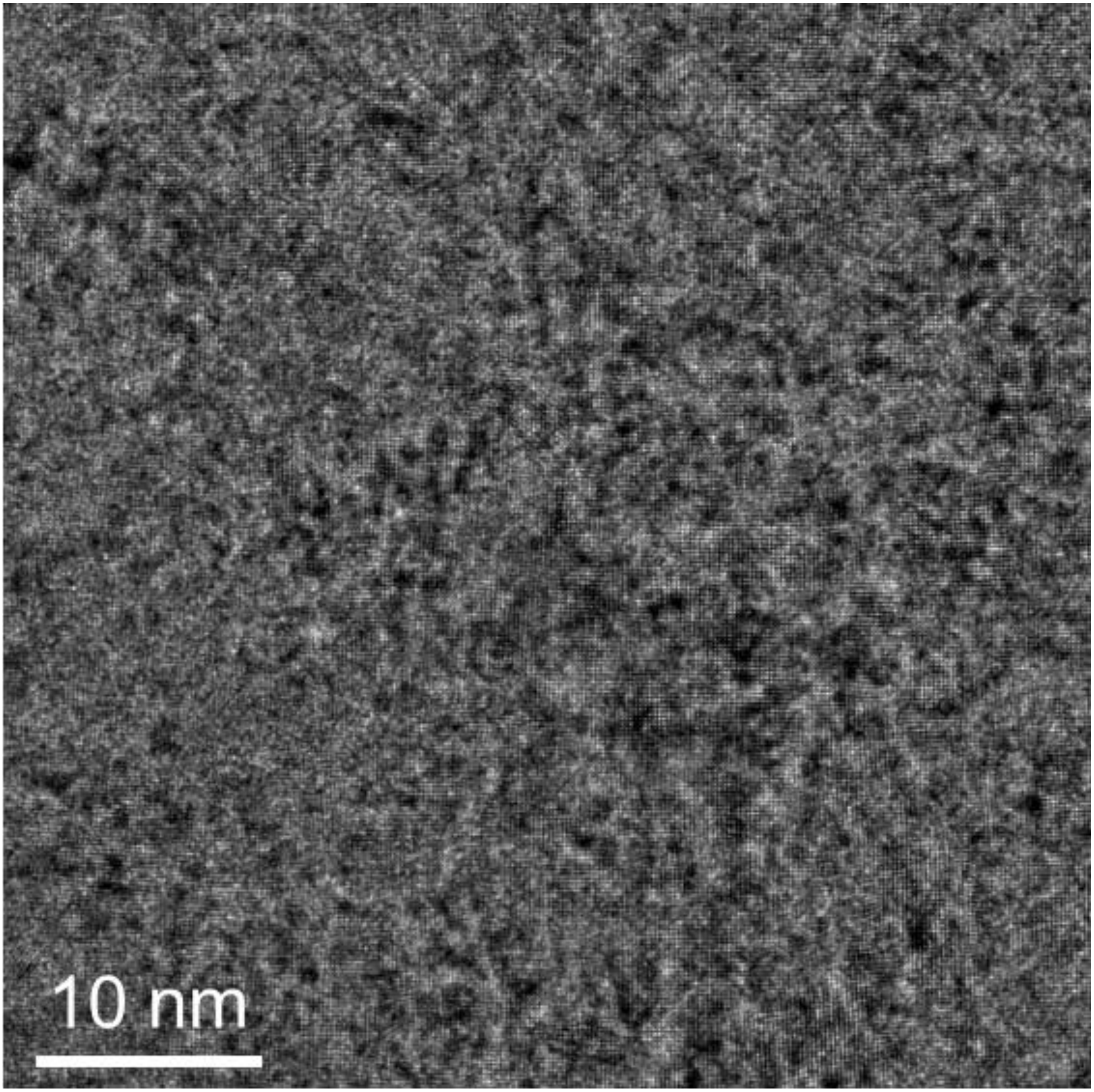

Supplementary Figure 23. The HRTEM image of the a sample after the 5th charging cycle.

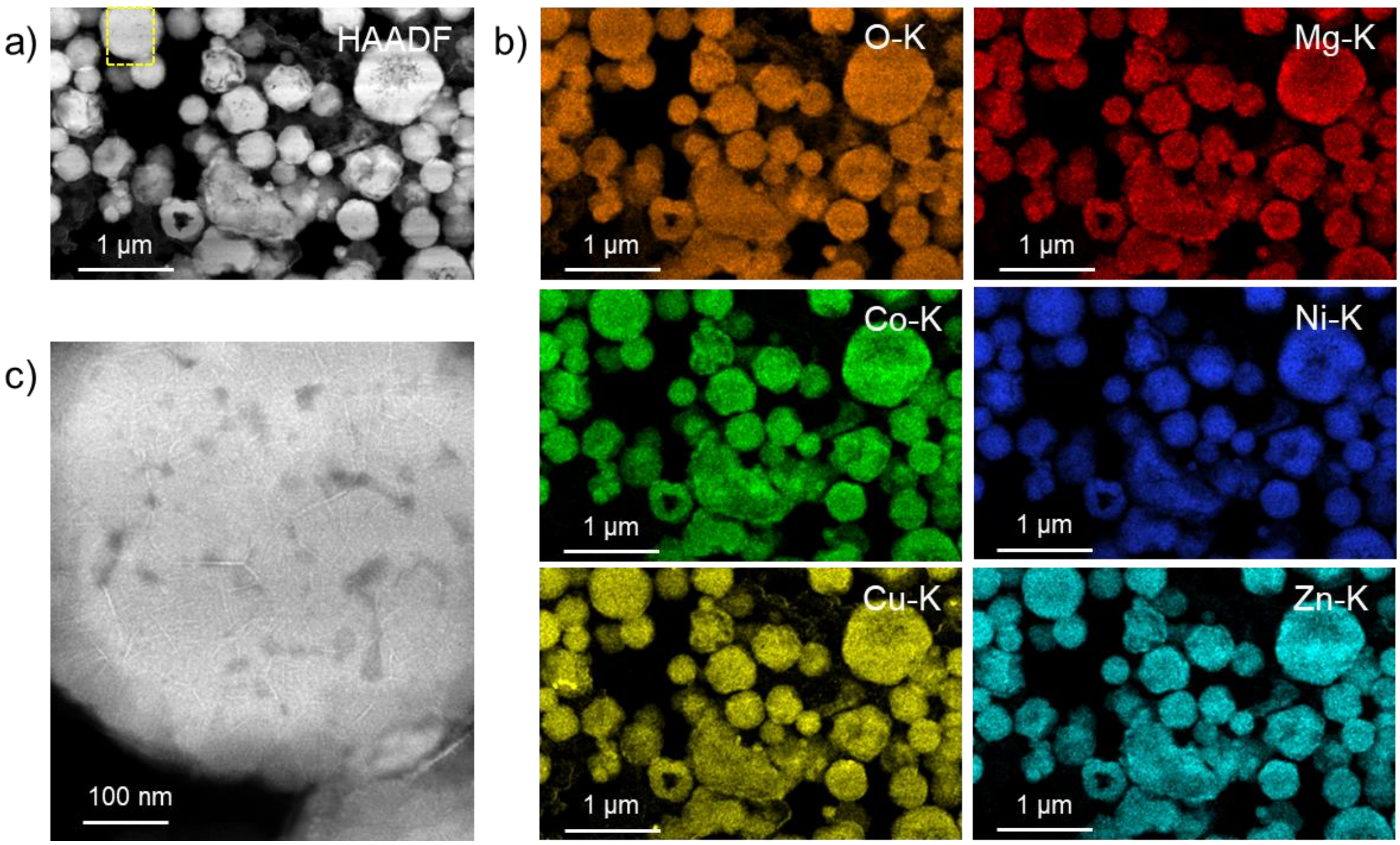

Supplementary Figure 24. Large area STEM-EDX map of HEO after 5 cycles in the charged state prepared by FIB. a) STEM-HAADF image, b) EDX maps and c) enlarged image of the area marked in a). No phase separation can be detected at this length scale. The pixel size of the map is 18.6 nm and field of view is 4.4 µm×2.8 µm.

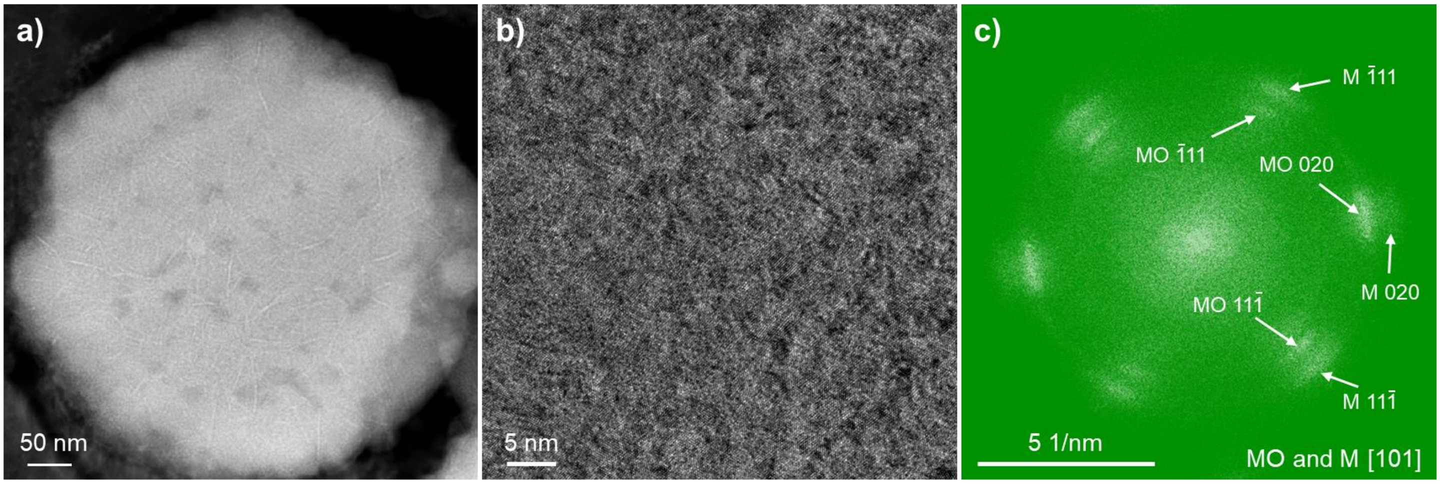

Supplementary Figure 25. (S)TEM analysis of a 5th cycled sample in the discharged stating showing very similar features as seen previously after the first discharge. a) STEM-HAADF image, b) HRTEM image and c) corresponding FFT.

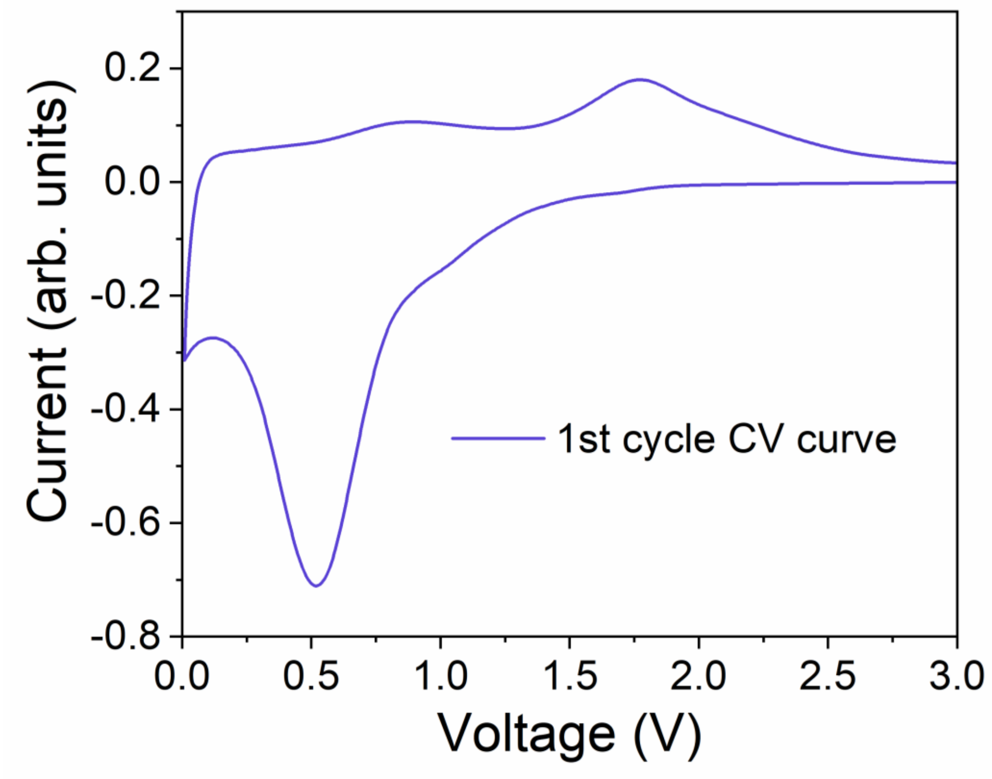

Supplementary Figure 26. The cyclic voltammetry (CV) curve of $Mg_{0.2}Co_{0.2}Ni_{0.2}Cu_{0.2}Zn_{0.2}O$ during the first cycle in the voltage window of 0.01 to 3.0 V. A very broad cathodic peak from 1.25 V to 0.2 V that presents the generation of SEI[6] and reduction of the transition metal (Cu[7], Co[8], Ni[9] and Zn[10]). The peak around 0.01V to 0.2 V is caused by the alloy reaction of Li_xZn ($x \leqslant 1$)[10]. In the subsequent delithiation process, the weak and broad peak below 1.5 V can be attributed to multi-step dealloying process of Li_xZn ($x \leqslant 1$) alloy.[10] The peak at around 1.5 to 2.5 V presents the oxidation process of Zn[10] and Co[8].

Reference

1. Hua, X. *et al.* Comprehensive Study of the CuF_2 Conversion Reaction Mechanism in a Lithium Ion Battery. *J. Phys. Chem. C* 118, 15169–15184 (2014).

2. Sarkar, A. *et al.* High entropy oxides for reversible energy storage. *Nat Commun* 9, 3400 (2018).

3. Dang, W. *et al.* Micro–nano NiO-$MnCo_2O_4$ heterostructure with optimal interfacial electronic environment for high performance and enhanced lithium storage kinetics. *Dalton Trans.* 49, 10994–11004 (2020).

4. Monari, S., Millo, D., Ranieri, A., Di, G. & Gert, R. The impact of urea-induced unfolding on the redox process of immobilised cytochrome c. *J Biol Inorg Chem* 15, 1233–1242 (2010).

5. Huang, X. H., Guo, R. Q., Wu, J. B. & Zhang, P. Mesoporous ZnO nanosheets for lithium ion batteries. *Materials Letters* 122, 82–85 (2014).

6. Liu, H., Zheng, Z., Chen, B., Liao, L. & Wang, X. Cobalt Oxide Porous Nanofibers Directly Grown on Conductive Substrate as a Binder / Additive-Free Lithium-Ion Battery Anode with High Capacity. *Nanoscale Research Letters* 12, 0–6 (2017).

7. Shi, L. *et al.* RSC Advances Synthesis of di ff erent CuO nanostructures from medium for lithium-ion battery anodes †. *RSC Adv.* 5, 28611–28618 (2015).

8. Jiang, J. *et al.* Direct Synthesis of CoO Porous Nanowire Arrays on Ti Substrate and Their Application as Lithium-Ion Battery Electrodes. *J. Phys. Chem. C* 114, 929–932 (2010).

9. Deng, W. & Chen, X. Graphitic carbon-wrapped NiO embedded three dimensional nitrogen doped aligned carbon nanotube arrays with long cycle life for lithium ion. *RSC Adv.* 8, 28440–28446 (2018).

10. Li, P. *et al.* Facile synthesis of ZnO/ mesoporous carbon nanocomposites as high-performance anode for lithium-ion battery. *CHEMICAL ENGINEERING JOURNAL* 271, 173–179 (2015).
