# Supplementary material for: Synergy of cations in high entropy oxide lithium ion battery anode
Source: Nat Commun. 2023 Mar 17;14:1487. doi: 10.1038/s41467-023-37034-6 (PMC10023782; doi:10.1038/s41467-023-37034-6)
Supplement: Supplementary file 3 — Description of Additional Supplementary Information [file 41467_2023_37034_MOESM3_ESM.pdf]

Description of Additional Supplementary Files

File Name: Supplementary Movie 1

Description: Tomographic reconstruction from the 1st charged HEO

File Name: Supplementary Movie 2

Description: Volume rendering of cropped tomo reconstruction

File Name: Supplementary Movie 3

Description: The generated video after surface rendering of the grain boundary in the marked area in Figure 6

File Name: Supplementary Movie 4

Description: The generated animation after combining the volume rendering of grain boundary area and the surface rendering of the grain boundary of the tomography data of 1st charged HEO
